# Supplementary material for: Yuanansuchus maopingchangensis sp. nov., the second capitosauroid temnospondyl from the Middle Triassic Badong Formation of Yuanan, Hubei, China
Source: PeerJ. 2016 Apr 5;4:e1903. doi: 10.7717/peerj.1903 (PMC4824913; doi:10.7717/peerj.1903)
Supplement: Appendix S1 [file peerj-04-1903-s001.docx]

**Appendix 1** Data line used in the cladistic analysis of capitosaurian relationships (Fig. 7). This represents two new lines added to the matrix presented by Fortuny et al. (2011). The character 50 of *Antarctosuchus* is changed to 1.

*Antarctosuchus* 0?0??012?0111201110?10?011011110??1111?????????111?11

*Y. laticeps* 0101101211010100100?00001110111?111111???????11100?20

*Y. maopingchangensis* 01021012011101101002000011101111?11111???????111???10

**Appendix 2** Character list

The following 57 characters were revised from the character lists of Schoch (2008) and Fortuny et al. (2011), both of them were derived from Damiani (2001). The following abbreviations are used here: D, Damiani (2001); S, Schoch (2008); F, Fortuny et al. (2011). The number following the abbreviation indicates the position of the character in the author’s list. Italics indicate that the definition of the character provided by the previous author(s) differs from that provided here.

1. **Preorbital region**. Parabolic (0), or tapering (1). [*D1*, S1, *F1*]

2. **Posterolateral skull corners (quadrates)**. Posterior to distal end of tabular horns (0), or anterior (1). [D2, S2, F2]

3. **Otic notch**. Deeply incised into posterior skull margin (0), or reduced to an embayment (1). [D3, S3, F3]

4. **Tabular horns**. Directed posteriorly (0), posterolaterally (1), or laterally (2). [*D4, S4, F4*] (This character is modified from previous works. Their laterally directed is really posterolaterally directed and is redefined as such here, and state 2 is a new state only present in *Yuanansuchus.*)

5. **Orbital margins**. Flush with plane of skull roof (0), or elevated above plane of skull roof (1). [D5, *S5*, F5]

6. **Postorbital-prepineal growth zone**. Absent (0), or present (1). [D6, S6, F6]

7. **Lateral line sulci**. Weakly impressed, discontinuous (0), or continuous, well impressed (1). [D7, S7, F7]

8. **Lacrimal flexure of infraorbital canal**. Absent (0), stepped (1), or Z-shaped (2). [D8, S8, F8]

9. **Occipital sensory canal**. Absent (0), or present (1). [D9, S9, F9]

10. **Supraorbital sensory canal**. Passes media to lacrimal (0), or enter lacrimal (1). [D10, *S10*, F10]

11. **Frontal**. Excluded from orbit (0), or entering medial margin of orbit. [D11, *S11*, F11]

12. **Supratemporal**. Entering dorsal margin of otic notch (0), or excluded from dorsal margin of otic notch (1). [D12, S12, F12]

13. **Preorbital projection of jugal**. Shorter than half the length of snout (0), or as long or longer (1). [*D13*, S13, *F13*]

14. **Postorbital**. Unexpanded (0), moderately antero-laterally expanded (1), or strongly anterolaterally expanded (2). [D14, *S14*, F14]

15. **Naris**. Oval (0), or narrow and elongated (1). [D15, S15, F15]

16. **Vomerine plate**. Short, as wide as long (0), or narrow and longer than wide (1). [*D16*, S16, *F16*]

17. **Occipital condyles**. Anterior to quadrate condyles (0), or level with or posterior to these (1). [D17, S17, F17]

18. **Choanal outline**. Oval-shaped (0), or narrow and slit-like (1), or circular (2). [D18, S18, F18]

19. **Transvomerine tooth row**. Transverse (0), or V-shaped (1). [D19, S19, F19]

20. **Anterior palatal vacuity**. Unpaired (0), or medially subdivided by anterior process (1), or completely subdivided (2). [*D20,* S20*, F20*]

21. **Pterygoid-parasphenoid suture**. As long as basal plate is wide (0), or substantially longer than basal plate is wide (1). [*D21*, S21, *F21*]

22. **Cultriform process extension between vomers**. Extends beyond anterior margin of interpterygoid vacuities (0), or underplated by vomers (1). [D23, S23, F22]

23. **Cultriform process**. Ventrally flat (0), or flat with central ventral ridge (1), or slender with deep ventral crest (“knife edged”) (2). [*D24,* S24*, F23*]

24. **Ectopterygoid**. Excluded from the lateral margin of interpterygoid vacuities (0), or entering margin, wedged between palatine and pterygoid (1). [*D25*, S25, *F24*]

25. **Crista muscularis, extension**. Behind posterior border of parasphenoid-pterygoid suture (0), or level with that border (1). [D26, S263, F25]

26. **Crista muscularis, midline**. Discontinuous (0), or confluent in midline (1). [D27, S27, F26]

27. **Parasphenoid pockets**. Facing posterodorsally, located along the posterior rim of the plate (0), or ventrally, entirely located on the flat surface (1). [D28, S28, F27]

28. **Exoccipital-pterygoid suture**. Absent (0), or present (1). [D29, S29, F28]

29. **Marginal teeth**. Circular or moderately oval (0), or anteroposteriorly compressed and closely set (1). [D30, S30, F29]

30. **Ectopterygoid tusks**. Present (0), or absent (1). [D31, S31, F30]

31. **Denticle field**. Present on parasphenoid and pterygoid (0), or absent (1). [D32, S32, F31]

32. **Quadratojugal**. Excluded from quadrate trochlea (0), or forming lateral portion of it (1). [D33, S33, F32]

33. **Posttemporal fenestra**. Narrow and slit-like (0), or triangular (1). [D35, S35, F33]

34. **Tabular horn**. Short, supported only by paroccipital process (0), or posteriorly extended, supported by two ventral ridges (1). [*D36*, S36, *F34*]

35. **Crista obliqua of pterygoid**. Absent (0), or present (1). [D37, S37, F35]

According to Schoch (2000), *Quasicyclotosaurus* has a ‘faintly developed crista oliqua’. Liu and Wang (2005) coded it as 0, but it is better coded as 1 as Schoch (2008). The coding for *Y. laticeps* is also changed as 1 here.

36. **Crista muscularis of parasphenoid**. Visible in occipital view (0), or not visible (1). [D38, S38, F36]

37. **Basioccipital**. Present (0), or absent (1). [D39, S39, F37]

38. **Crista falciformis**. Absent (0), or present (1). [D40, S40, F38]

39. **Postglenoid area**. Short boss (0), or distinct process (1), or extended, longer than glenoid facet (2). [*D41*, S41, *F39*]

40. **Hamate process of prearticular**. Absent or rudimentary, forming at best an anterior margin of the glenoid facet (0), or present, raised well above glenoid and as high as quadrate trochlea (1), or substantially higher than quadrate trochlea (2). [*D42*, S42, *F40*]

41. **Posterior Meckelian fenestra**. Small and round (0), or elongated (1). [D43, S43, F41]

42. **Labial wall of adductor chamber**. Dorsally horizontal (0), or dorsally convex (1). [D44, S44, F42].

43. **Coronoid series**. With tooth patch (0), or single row of teeth (1). [D45, S45, F43]

44. **Prearticular**. Sutures with splenial anteriorly (0), or separated from it by dentary or coronoid 2 (1). [*D46*, S46, *F44*]

45. **Glenoid facet**. Above level of dorsal surface of dentary (0), or below (1). [D47, S47, F45]

46. **Parietals and supratemporal length.** The parietal longer than the supratemporal (0), or similar in length (1). [*F46*]

47. **Postparietals and tabular length**. Shorter than parietals (0), or similar (1). [S53*, F47*]

48. **Interpterygoid vacuities**. Equally wide anteriorly and posteriorly (0), or basipterygoid ramus of the pterygoid is medially thickened and constricts the interpterygoid vacuities (1). [*S59,* F48]

49. P**osterior margin of the squamosal.** Straight or curved in a slightly convex manner (0), or forming a posteriorly extended plate that roofs the occiput (1). [F50]

50. **Postglenoid area**. Oblique dorsal and ventral margins (0), or straight dorsal margin bilaterally (1). [*F51*]

51. **Skull greatest width−to−midline length in adults**. 0.8–1.2 (0), <0.8 (1), >1.2 (2). [F52]

52. **Postorbital and prefrontal**. Widely separated (0), or nearing one another by thin projections (1), or sutured, excluding jugal from orbit margin (2). [S58, F53]

**53. Otic fenestra**. Tabular and squamosal separated by otic notch posteriorly (0), or separated by narrow slit (1), or sutured to encircle an otic fenestra (2). [S54].

54. **Palatine ramus of pterygoid**. Ventrally smooth (0), or ornamented (1). [S49]

**55. Posterior boss of quadrate**. Occipital face of quadrate with prominent posterior boss (0), or smooth (1). [S62]

56. **Cultriform process**. Merges continuously from basal plate (0), or forming a deltoid base (1). [S51]
